# New Frontiers for the Early Diagnosis of Cancer: Screening miRNAs Through the Lateral Flow Assay Method

**DOI:** 10.3390/bios15040238

**Published:** 2025-04-08

**Authors:** Afsaneh Emamiamin, Seyedeh Rojin Shariati Pour, Thea Serra, Donato Calabria, Marta Varone, Fabio Di Nardo, Massimo Guardigli, Laura Anfossi, Claudio Baggiani, Martina Zangheri, Mara Mirasoli

**Affiliations:** 1 Department of Chemistry “Giacomo Ciamician”, Alma Mater Studiorum, University of Bologna, Tecnopolo di Rimini, Via Dario Campana 71, I-47922 Rimini, Italy; afsaneh.emamiamin2@unibo.it (A.E.); seyedeh.shariatipou3@unibo.it (S.R.S.P.); 2 Department of Chemistry, University of Turin, Via P. Giuria 5, I-10125 Turin, Italy; thea.serra@unito.it (T.S.); fabio.dinardo@unito.it (F.D.N.); laura.anfossi@unito.it (L.A.); claudio.baggiani@unito.it (C.B.); 3 Department of Chemistry “Giacomo Ciamician”, Alma Mater Studiorum, University of Bologna, Via Francesco Selmi 2, I-40126 Bologna, Italy; donato.calabria2@unibo.it (D.C.); marta.varone2@unibo.it (M.V.); massimo.guardigli@unibo.it (M.G.); 4 Interdepartmental Centre for Industrial Aerospace Research (CIRI AEROSPACE), Alma Mater Studiorum—University of Bologna, Via Baldassarre Canaccini 12, I-47121 Forlì, Italy; 5 Interdepartmental Centre for Industrial Research in Renewable Resources, Environment, Sea, and Energy (CIRI FRAME), Alma Mater Studiorum—University of Bologna, Via St. Alberto 163, I-48123 Ravenna, Italy; 6 Interdepartmental Centre for Industrial Agrofood Research (CIRI AGRO), Alma Mater Studiorum—University of Bologna, Via Quinto Bucci 336, I-47521 Cesena, Italy

**Keywords:** lateral flow assay, point-of-care, microRNA, biosensor, cancer

## Abstract

MicroRNAs (miRNAs), which circulate in the serum and plasma, play a role in several biological processes, and their levels in body fluids are associated with the pathogenesis of various diseases, including different types of cancer. For this reason, miRNAs are considered promising candidates as biomarkers for diagnostic purposes, enabling the early detection of pathological onset and monitoring drug responses during therapy. However, current methods for miRNA quantification, such as northern blotting, isothermal amplification, RT-PCR, microarrays, and next-generation sequencing, are limited by their reliance on centralized laboratories, high costs, and the need for specialized personnel. Consequently, the development of sensitive, simple, and one-step analytical techniques for miRNA detection is highly desirable, particularly given the importance of early diagnosis and prompt treatment in cases of cancer. Lateral flow assays (LFAs) are among the most attractive point-of-care (POC) devices for healthcare applications. These systems allow for the rapid and straightforward detection of analytes using low-cost setups that are accessible to a wide audience. This review focuses on LFA-based methods for detecting and quantifying miRNAs associated with the diagnosis of various cancers, with particular emphasis on sensitivity enhancements achieved through the application of different labels and detection systems. Early, non-invasive detection of these diseases through the quantification of tailored biomarkers can significantly reduce mortality, improve survival rates, and lower treatment costs.

## 1. Introduction

Cancer is a leading cause of death worldwide, accounting for nearly 10 million deaths in 2020, or nearly one in six deaths [1]. The high mortality is due to the lack of effective early detection methods for these malignancies. Indeed, cancer is a silent disease, and its early stages are difficult to detect because most symptoms are nonspecific and thus of little use in diagnosis. As a result, it is rarely identified until it spreads and advances to later stages, and the cancer has already diffused.

Therefore, the battle against cancer should be conducted not only with intensive research on new effective treatments, but it should also be mainly based on the development of innovative diagnostic methods that allow the prompt detection of the onset of the disease. In this context, the fundamental aspect that would lead to a great breakthrough in the fight against cancer is the possibility to perform screening very frequently.

To promote frequent screenings, they should be based on non-invasive methods involving the use of untreated biological samples that can be collected easily and painlessly.

Diagnosis generally requires invasive and stressful approaches involving physical examination. Concerning blood biomarkers, the measurement of different serum cancer antigens can be exploited. However, they display low specificity and sensitivity, and therefore, the European Group on Tumor Markers and the National Academy of Clinical Biochemistry do not recommend using these molecules as biomarkers for the early diagnosis of cancer [2]. Therefore, researchers must focus on the possibility of finding new biomarkers in body fluids that can be much more predictive for these malignancies.

In this context, microRNAs (miRNAs) are strong candidates as potential diagnostic markers for different pathological conditions [3]. miRNAs are small (typically 18–25 nucleotides in size) regulatory RNA molecules involved in essential biological processes that have been observed to be abnormally expressed in different diseases, especially in cancers [4]. 

Since the discovery of circulating miRNAs [5,6], there has been significant interest in identifying and validating panels of these miRNAs associated with cancer onset and progression. These biomarkers are stable, resistant to RNase activity, and could enable cancer diagnosis using a simple blood sample instead of a more invasive biopsy [7,8,9,10]. Different kinds of tumors have different miRNA expression spectra, thus providing higher specificity than conventional cancer biomarkers, and their expression profiles significantly change even in the early stage of cancer, thus allowing early diagnosis [11]. Although miRNAs have been proposed as biomarkers for cancer diagnosis, their application in routine clinical practice is yet to come [12].

Indeed, one of the biggest problems in detecting miRNAs is their small size, which makes it difficult to design a specific probe for binding the target miRNA of interest [13], thus resulting in a high rate of false positives [14].

Current quantification strategies, like Northern blot, microarrays, quantitative real-time polymerase chain reaction, in situ hybridization, and high-throughput sequencing, have limitations, as they require complex sample pre-treatment steps and specialized personnel and centralized laboratories, thus increasing assay time and cost [15,16]. Indeed, another aspect that could encourage large-scale screening is the low cost of the method itself, which would allow access to frequent control to a greater part of the population. The reduction in cost involves two fundamental aspects. The first is the possibility of carrying out these analyses outside of centralized laboratories by non-specialized personnel, thus involving the development of portable and disposable analytical devices that are easy to use and affordable for everyone. Consequently, the second aspect is related to the materials and platform used for performing the analysis, which must be very simple, easy to handle, and widely available on the market.

Combining these aspects, the development of an analytical device that allows the ultra-sensitive detection of blood biomarkers highly predictive for early stages of cancer through the use of a minimally invasive test that can be performed by untrained personnel would enable large-scale screening at a low cost. Indeed, this method could be used directly in the medical office or by the patients themselves in order to perform periodic checks for early detection of the onset of this disease. The point-of-care (POC) approach would allow early detection of cancer, enabling timely execution of a confirmation test and, if necessary, prompt activation of adequate therapies, greatly increasing the rate of therapy success and thus the chances of survival.

The development of an accurate and user-friendly diagnostic device for POC detection of specific miRNAs is one of the most challenging objectives in the clinical and medical chemistry field [17,18]. Devices for POC should be portable, rapid, and easy to use, and they should enable the entire analytical process to be performed in a non-laboratory environment. The ASSURED criteria, as defined by the World Health Organization, are based on the fact that POC devices must be characterized by several features, including affordability, sensitivity, specificity, user-friendliness, rapidity, and robustness; they must also be equipment-free and delivered to the end users. Within this context, the technology of lateral flow-based assays (LFAs), widely known for commercial pregnancy tests, is considered one of the most feasible and commercially successful analytical tools for rapid and portable clinical diagnostics. This method is based on the use of an assembly of a nitrocellulose strip and one or more cellulose-based pads, each serving one or more purposes. The components overlap and are affixed to a backing card using a pressure-sensitive adhesive. During testing, the sample is applied to the strip’s proximal end, known as the sample pad, where it undergoes treatment to ensure compatibility with the rest of the assay. The treated sample then moves to the conjugate pad, which contains an immobilized particulate conjugate. This conjugate is linked to a specific biological component of the assay, depending on the assay format. As the sample progresses, it rehydrates the dried conjugate, allowing the analyte to interact with it while both migrate into the reaction matrix. This matrix consists of a porous nitrocellulose membrane, where the assay’s other specific biological component is immobilized at designated locations. These areas capture the analyte at the test line and the conjugate at the control line as they continue migrating through the strip. Excess reagents move past the capture lines and are entrapped in the absorbent pad. Results are interpreted on the reaction matrix as the presence or absence of lines of captured conjugate, read either by eye or using a specifically developed reader [19]. The test is completed in a few minutes, exploiting capillary forces to ensure the flow of samples and reagents.

**Figure 1:**
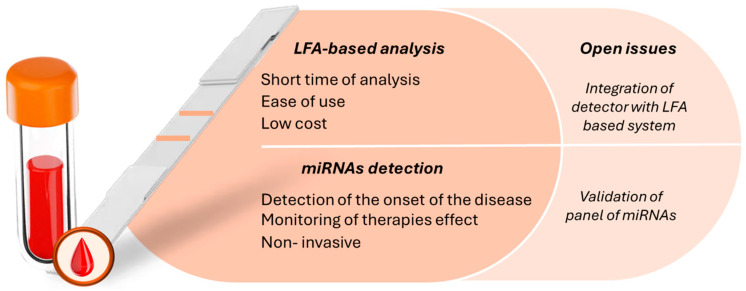
Schematic representation of advantages and open issues in coupling LFA-based methods with miRNA quantification applied to cancer diagnosis.

Although LFAs are usually based on immunoassays, they have also been developed for nucleic acids and, more recently, for miRNA detection [13,20,21,22] (Figure 1).

The main limitation of conventional LFAs is that they only provide a “yes/no” qualitative response on the analyte’s presence or absence, as detection is based on the formation of colored bands that are visually detected on the nitrocellulose strip due to the use of gold nanoparticles (GNPs) [23] and latex microbeads [24,25,26,27] as detection labels. Recently, tremendous research has been carried out to provide novel and efficient tags suitable for increasing the sensitivity of the method. In particular, GNPs have been replaced by tracers based on electrochemical, fluorescence, chemiluminescence, SERS, magnetism, and thermal contrast in order to achieve ultrasensitive quantitative information [28,29,30]. Among the most innovative proposed tags, fluorescent nanoparticles have attracted a lot of interest, as it is possible to exploit a very wide range of materials, such as quantum dots [31], sequences labeled with fluorescent moieties [32], dye-doped silica nanoparticles [33], and up-conversion luminescent materials [34].

Also, nanomaterials have been extensively proposed thanks to the possibility of amplifying the detection signal, thus obtaining high sensitivity for the detection of the target analytes [35,36]. In this context, exploiting nanotechnologies, a great variety of signal amplification strategies have been applied to lateral flow assays, including increasing the specific surface area of the signal tag by surface modification of materials (e.g., silica, gold, and silver) [37]; enzyme-linked labeling (e.g., GNPs loaded with large amounts of horseradish peroxidase (HRP)) to amplify the reaction process [20]; and auxiliary signal amplification using DNA nanomachines (molecular amplification techniques, such as hybridization chain reaction (HCR)), rolling circle amplification (RCA), and catalyzed hairpin assembly (CHA) [38,39,40]. Furthermore, some structural modifications of LFAs have been proposed in order to allow the performance of multiplex analysis [41,42]. Indeed, the most accurate clinical diagnosis is generally based on a set of information that, linked together, offers a precise clinical framework [17]; thus, the simultaneous detection of a biomarker panel is much more predictive compared to the use of just a single biomarker. The aim of this review is to present a state-of-the-art overview of the proposed analytical devices based on the LFA method for the detection of miRNAs that may be useful for the diagnosis of cancer. The review focuses on the different strategies adopted for the quantification of target analytes, including the different detection and amplification approaches of the analytical signal.

## 2. Colorimetric LFA: A Traditional Approach Applied to Detection of miRNA as a Cancer Biomarker

### 2.1. GNPs Exploited as Colorimetric Label for Visual Detection

The dominant strategy exploited in the LFA approach is colorimetric detection based on the use of colored labels to obtain both qualitative information through the naked eye and, when necessary, quantitative measurements by integrating the device with a suitable reader.

Most works report the use of GNPs as labels, as these are particularly advantageous in terms of the simplicity of conjugation to biospecific probes and stability [43,44]. In these cases, to increase the detectability of the target analyte by the naked eye through the coloring of the test line, an amplification step is carried out before the LFA test. In this context, various amplification strategies and different approaches for target recognition have been tested.

Yao et al. [40] reported on the combination of rolling circle amplification (RCA) and GNP-based LFA for multiplex detection of miRNA 21 and miRNA let-7a. The amplification step played an essential role in increasing sensitivity, allowing the specific and selective detection of both target analytes, showing limits of detection (LOD) of 20 pM (miR-let-7a) and 40 pM (miR-21). Also, Zhang et al. [45] exploited RCA to detect exosomal 5-methylcytosine miRNA-21 (m5C-miRNA-21). In this case, RCA amplification was triggered by DNAzyme for recognizing and cleaving m5C-miRNA-21 in the presence of Mg^2^+. A padlock DNA was designed based on one of the cleaved strands of m5C-miRNA-21 as the primer, and the RCA reaction occurred under the action of T4 ligase and DNA polymerase. In LFA-based assays, RCA products are cut by nicking endonuclease (Nb.BbvcI) into repeating nucleic acid fragments (RCA fragments). Then, these RCA fragments are added onto the conjugate pad of the LFA device and combined with GNP–DNA probes to form RCA fragments/GNP–DNA complexes. Flowing over the test line, these complexes hybridize with the immobilized probe, resulting in a color band visible to the naked eye.

Kim et al. [46] proposed a multiplex approach in which they integrated reverse transcription using a stem-loop primer with a DNA barcode-based LFA for detecting miR-92a and miR-141. The assay was designed in order to enable the binding of the single-stranded PCR products obtained in the presence of the target miRNA to capture DNA immobilized on the nitrocellulose strip.

Similarly, Gao et al. [47] reported on an LFA based on sandwich DNA–miRNA–DNA/GNPs for detecting miR-215. The capture probe was immobilized on the test line, exploiting the binding between biotin and streptavidin. The detection probe was labeled with GNPs and stored on the conjugate pad. When the target was present, it bound to both the detection and capture probes, forming a sandwich-like structure. This assay did not require an amplification step.

The same approach was proposed by Zheng et al. [48], in which the principle of the LFA was based on sandwich-type hybridization reactions to produce ssDNA–miRNA-ssDNA/GNP complexes, which were captured and visualized on the test zones to detect miR-21, miR-155, and miR-210 simultaneously.

**Figure 2:**
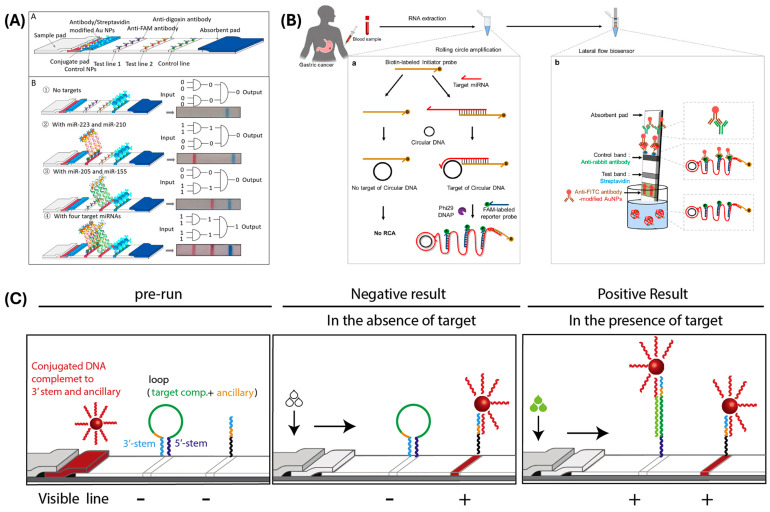
(**A**) Scheme of the LFA-based assay. (**B**) Readout based on DSN-mediated and AND logic gate. Reprinted with permission from Ref. [49]. Copyright 2023 American Chemical Society. (**B**) Scheme of the isothermal amplification-based LFA. (**a**) RCA-based isothermal amplification reaction for miRNA detection in blood samples (B: biotin; F: FAM fluorescent dye). (**b**) The amplified RCA product binds to antibody-modified GNPs, and the target miRNA can be visually identified. Reprinted with permission from Ref. [52]. (**C**) Scheme of the assay principle for MB-BSH LFA. Conjugated DNA complementary to the 3′ stem and ancillary sequence in the loop of MB compete with the 5′ stem for binding to the 3′ stem. The MBs switch their structures in the presence of the target. Reprinted with permission from Ref. [54].

Another example of a multiplex approach involving an amplification step was described by Zhou et al. [49]. In particular, they developed duplex-specific nuclease (DSN)-mediated signal amplification combined with dual-AND logic gate-based signal output for the detection of four miRNAs. Different from the other cases, the authors proposed an LFA strip in which antibodies were used to detect the miRNAs of interest. In particular, during the amplification step, the target analytes were labeled with biotin, digoxin, FAM, and TAMRA, which were captured by their respective antibodies immobilized on the test lines (Figure 2A).

The same LFA approach exploiting antibodies on the test line instead of DNA-based probes was used by Hou et al. [50], who proposed the formation of an avidin–biotin–GNP–target analyte complex captured by anti-avidin antibodies immobilized on a nitrocellulose strip to detect miR-21. When there was no miR-21 in the samples, in order to avoid non-specific signals, mung bean nuclease, a single-strand-specific nuclease, catalyzed the degradation of the capture probe.

Also, Lamprou et al. [51] introduced a universal LFA exploiting antibodies instead of DNA-based probes, combined with reverse transcription polymerase chain reaction (RT-PCR) and GNPs as labels, for the detection of miR-21 and miR-let-7a in human urine. During RT-PCR, the target analytes were biotinylated. The GNPs on the conjugate pad were conjugated to anti-biotin antibodies that recognized the amplification products, while on the test lines, beads conjugated with detection probes complementary to a part of the amplification products were immobilized.

Seo et al. [52] reported on a diagnostic platform based on the combination of RCA with the LFA approach for detecting miR-135b and miR-21. During the RCA step, the target analytes were labeled with biotin and a fluorescent dye. When the amplification products were injected onto the LFA strip, they bound to anti-FITC antibodies labeled with GNPs on the conjugate pad, and these complexes then were captured by streptavidin immobilized on the test lines (Figure 2B).

As an alternative to antibodies, the use of a molecular beacon (MB) was proposed by Kor et al. [53], who designed biotin–MB–GNPs to modulate the accessibility of the biotin group as a function of the presence of an miRNA target, allowing interaction with the streptavidin immobilized on the test line. When the target miRNA is present, the MB, which is bound to GNP and biotin at opposite ends, opens its structure, allowing the biotin to bind streptavidin on the test line.

Also, Javani et al. [54] exploited the peculiar characteristic of the MB structure to develop an LFA-based assay for the detection of miR-210 and miR-424. The authors proposed a design for an LFA based on the switching of the MB in the presence of the target analytes without the use of streptavidin or any other affinity protein. GNPs were used as labels for complementary detection probes selected for recognizing the analytes of interest. These complexes were then captured by the MB immobilized on the test lines (Figure 2C).

MBs were also used by Huang et al. [55], who presented a system composed of an MB probe, an assistant probe, and an endonuclease for amplifying the analytical signal. In the presence of the miRNA of interest, a Y-shaped junction structure was formed upon the hybridization of three nucleic acid chains. This structure could be recognized by the endonuclease. The MB probe was then cleaved by the endonuclease, thus producing two new DNA fragments, while the regenerated assistant probe and target were hybridized to another MB probe and entered the next cycle of amplification. Following this procedure, the initial miRNA was converted into two DNA fragments that could be detected using the LFA approach, in which two test lines were designed to detect both DNA fragments resulting from the amplification step. The GNPs were linked to a specific DNA sequence, and on the test lines, DNA capture probes were immobilized, forming a sandwich-like structure when the target analyte was present. Endonuclease was also exploited by Peng et al. [56], who reported on the combination of the LFA technique with an amplification step based on the self-primer exponential amplification reaction (SPEXPAR). The colorimetric LFA was designed in order to detect the amplification products, which were labeled with a fluorescent dye (FAM). The GNPs were linked to anti-FAM antibodies to obtain a complex that was then recognized by the DNA probe immobilized on the test line.

A similar approach was reported by Ying et al. [57]. The authors combined duplex-specific nuclease (DSN) and HCR in order to perform a preliminary step in which the target analyte was amplified and conjugated to FAM and biotin. In this way, during the LFA-based analysis, the amplification products bound to the GNPs conjugated to streptavidin, forming a visible complex that could be detected at the test line, on which the anti-FAM antibodies were immobilized.

Wang et al. [58] exploited catalytic hairpin assembly to enable sensitive detection of miR-21. In particular, the presence of the target analyte triggered the self-assembly of two hairpin DNAs into double-stranded DNA, exposing biotin molecules on the surface of GNPs. The GNPs carrying biotin were captured on the test line of the strip, allowing the formation of a visible red band.

Recently, Xu et al. [59] integrated a tetrahedral probe and catalytic hairpin assembly. DNA tetrahedrons were labeled with biotin to act as capture probes on the test line. The catalytic hairpin assembly system consisted of two hairpin sequences that could form double-stranded products through an assembly reaction triggered by the target analyte. The duplexes hybridized with barcoded tetrahedra exploiting complementary sequences and bound to streptavidin-modified GNPs via biotin.

An innovative approach for selectively detecting a specific miRNA is based on the use of clustered regularly interspaced short palindromic repeats (CRISPR) and their CRISPR-associated (Cas) protein strategy. Indeed, CRISPR/Cas technologies represent a promising tool in molecular diagnostics thanks to their suitability for sequence-specific identification [60,61]. In particular, the possibility of obtaining target-activated trans-cleavage activity of Cas12, Cas13, and Cas14a, which effectively and indiscriminately cleave single-stranded DNA or single-stranded RNA, makes them applicable for signal-amplified nucleic acid detection [62,63,64]. In the field of LFA development for miRNA detection, few examples have been reported in the literature. 

Tian et al. [65] reported a tandem CRISPR/Cas13a/Cas12a mechanism coupled with an LFA-based analysis for the detection of miR-21. Specific RNA sequences were designed to guide Cas12a and Cas13a to their respective targets: miRNA-21 and unlocked DNA. The locked RNA/DNA complex, containing unRNA and unDNA, served as both a substrate for Cas13a and a site-specific target for Cas12a. In the presence of miRNA-21, the unlocked RNA within the locked RNA/DNA complex was degraded due to the trans-cleavage activity of Cas13a, leading to the release of unDNA. This free unDNA then acted as a target sequence, triggering the trans-cleavage activity of Cas12a. As a result, the biotin–ssDNA–FAM reporters were efficiently cleaved by the activated Cas12a, causing the separation of biotin from FAM. This process disrupted the sandwich structure of GNP@anti-FAM-Ab/FAM-ssDNA-Bio/SA, allowing the excess GNP@anti-FAM-Ab on the control line to migrate to the test line, where it was recognized by the anti-mouse antibody.

In conclusion, as shown in Table 1, the use of GNPs as colorimetric labels is widespread because of their high stability and ease of conjugation with biospecific probes. The signal is then detected either visually or by integrating a detector capable of providing quantitative information dependent on the intensity of the coloration. However, the intrinsic sensitivity of these systems is often limited, making amplification strategies necessary for the accurate detection of low-concentration miRNAs. The integration of amplification techniques such as RCA has shown significant improvements in test sensitivity, and different amplification approaches have been tested. Further differences between the reported works are related to the immobilization strategy for capture probes on the T-lines, as well as for the conjugation of GNPs to specific detection probes. Indeed, innovation is represented by the use of MB probes that modulate detection based on the presence of target miRNAs, as well as the use of CRISPR/Cas-based technologies, as described by Tian et al., who utilized tandem Cas13a/Cas12a mechanisms. These advancements not only improve sensitivity and specificity but also broaden the potential applications of LFA in cancer biomarker diagnostics.

**Table 1:** LFA-based biosensing systems for the quantification of miRNAs based on the use of GNPs as labels.

Method Principle	Lateral Flow Assay	Key Strengths	Weaknesses and Areas Needing Improvement	Biomarker	LOD	Ref.
Combination of rolling circle amplification and GNP-based LFA for multiplex detection of miRNA.	Two DNA-based probes (one immobilized on the test line, one labeled with GNPs) form a sandwich structure with RCA products.	Multiplex strategy, only one capturing probe was needed	Amplification step is required	miR-let-7a miR-21	20 pM (miR-let-7a) and 40 pM (miR-21)	[40]
The RCA amplification was initiated by DNAzyme, which specifically recognized and cleaved the target analyte in the presence of Mg^2+^. A padlock DNA, designed using one of the cleaved strands of m5C-miRNA-21 as the primer, facilitated the RCA reaction driven by T4 ligase and DNA polymerase.	The resulting RCA products were then cleaved into repeating nucleic acid fragments using a nicking endonuclease. These fragments were subsequently applied to an LFA strip, where they formed complexes with GNP–DNA probes. These complexes hybridized with bio-ssDNA-1 immobilized on the test line.	High specificity due to DNAzyme triggered RCA reaction	Amplification step is required	m5C-miRNA-21	0.1 pM	[45]
Multiplex detection of miRNAs obtained by coupling reverse transcription using a stem-loop primer with DNA barcode-based LFA.	PCR primers with a DNA barcode sequence were designed to hybridize to capture DNA immobilized on the nitrocellulose membrane.	Multiplex detection of exosomal miRNAs exploiting DNA barcode technology	Amplification step is required	miR-92a and miR-141	Not reported	[46]
LFA based on sandwich DNA–miRNA–DNA/GNPs.	The capture probe was anchored to the test line via the interaction between biotin and streptavidin. The detection probe, labeled with GNPs, was preloaded onto the conjugate pad. In the presence of the target, it bound to both the detection and capture probes, forming a sandwich-like structure. This assay functioned without the need for an amplification step.	The biosensor was applied to cell lysate directly without complex sample treatment	Improvements in the sensitivity could be achieved using HRP–GNP dual labels and the detection of mismatched miRNA	miR-215	60 pM	[47]
Multiplex approach based on sandwich-type hybridization reactions without the use of an amplification step.	Sandwich-type hybridization reactions to produce ssDNA–miRNA–ssDNA/GNP complexes, which were captured and visualized in the test zones.	Multiplex approach, no amplification step required	Further work could improve the sensitivity and test the biosensor in serum samples from patient and healthy controls	miR-21, miR-155, and miR-210	0.007 nM (miRNA-155), 0.068 nM (miRNA-21), 0.017 nM (miRNA-210)	[48]
Duplex-specific nuclease (DSN)-mediated signal amplification combined with dual-AND logic gate-based signal output for the detection of four miRNAs.	During the amplification step, the target analytes were labeled with biotin, digoxin, FAM, and TAMRA, which were captured by their respective antibodies immobilized on the test lines.	Multiplex approach based on logic gate method	Amplification step is required	miR-223 miR-210 miR205 miR-155	26.51 fM	[49]
Universal LFA exploiting antibodies instead of DNA-based probes combined with reverse transcription polymerase chain reaction (RT-PCR) and GNPs as labels.	During the RT-PCR process, the target analytes were biotinylated. The GNPs on the conjugate pad were linked to anti-biotin antibodies, which recognized the amplification products. Meanwhile, detection probes complementary to a portion of the amplification products were immobilized on the test lines, conjugated to beads.	Multiplex approach; the approach is based on the use of antibodies as probe to obtain a universal tool easily adaptable to the detection of other miRNAs	Amplification step is required; in the future, detection could be applied to miRNAs with single-base variations	miR-21 and miR-let-7a	10^2^–10^3^ copies (miR-21), 10^2^–10^4^ copies (miR-let-7a)	[51]
Combination of RCA and LFA based on the use of biospecific proteins for recognizing amplification products.	During the RCA step, the target analytes were labeled with biotin and a fluorescent dye. Upon introducing the amplification products onto the LFA strip, they bound to anti-FITC antibodies conjugated with GNPs on the conjugate pad. These complexes were subsequently captured by streptavidin immobilized on the test lines.	Multiplex approach, the test was applied to samples from real patients	Amplification step is required	miR-135b and miR-21	0.1 nM	[52]
Biotin–MB–GNPs were designed to regulate the accessibility of the biotin group based on the presence of an miRNA target, enabling interaction with streptavidin immobilized on the test line.	When the target miRNA was present, the MB, attached to GNP and biotin at opposite ends, underwent a structural change, allowing the biotin to bind to streptavidin on the test line.	The use of an MB as a probe makes the assay less sensitive to the experimental condition, which is particularly important in the case of short targets such as miRNA	The approach could be extended to the detection of other miRNAs by tuning the sequence of the MB	miR-21	115 pM	[53]
A universal GNP DNA conjugate was hybridized through the base stacking hybridization (BSH) phenomenon to bind a specific MB on the test line.	GNPs served as labels for complementary detection probes designed to recognize the target analytes. These complexes were subsequently captured by the MB immobilized on the test lines.	Multiplex assays were developed with a unique GNP–DNA probe	Signal improvement methods (i.e., biotinylation of capture DNAs, application of affinity proteins, utilization of HRPs at the surface of GNPs) could be applied	miR-210 and miR-424	10 pmol for both miRNAs	[54]
The amplification step involves a system consisting of an MB probe, an assistant probe, and an endonuclease to enhance the analytical signal.	In the presence of the target miRNA, a Y-shaped junction structure is formed through the hybridization of three nucleic acid strands, which is recognized by the endonuclease. The MB probe is cleaved by the endonuclease, generating two new DNA fragments. The regenerated assistant probe and target miRNA then hybridize with another MB probe, entering a new cycle of amplification. Through this process, the initial miRNA is converted into two DNA fragments, which can be detected using the LFA approach. Two test lines are designed to detect each DNA fragment produced during amplification. GNPs are conjugated to specific DNA sequences, and DNA capture probes are immobilized on the test lines, forming a sandwich-like structure in the presence of the target analyte.	The enhancement ability of endonuclease-assisted target recycling amplification allows signal translation and amplification	Amplification step is required	miR-16	0.1 pM	[55]
Combination of the LFA technique with an amplification step based on the self-primer exponential amplification reaction (SPEXPAR).	The colorimetric LFA was designed to detect amplification products labeled with a fluorescent dye (FAM). GNPs were conjugated with anti-FAM antibodies to form a complex, which was subsequently recognized by the DNA probe immobilized on the test line.	The biosensor was applied to human serum samples with good recoveries	Amplification step is required	miR-155	100 pM	[56]
Before LFA-based analysis, an amplification step was performed combining DNS and HCR approaches.	The amplification products were conjugated to both biotin and FAM. During the LFA-based analysis, the amplification products bound to GNPs conjugated with streptavidin, forming a visible complex. This complex was detected on the test line, where anti-FAM antibodies were immobilized.	Dual signal amplification increases sensitivity and lowers the limit of detection	Amplification step is required	miR-21	2.1 fM	[57]
Hairpin 1, modified with biotin, was immobilized on GNPs. Due to the hairpin structure, the biotin molecule on the H1 probe is in close proximity to the GNPs, preventing the probe from being captured by streptavidin on the LFA strip. In the presence of microRNA-21 and Hairpin 2 (H2), CHA amplification is triggered on the GNPs, producing multiple double-stranded DNA molecules that expose biotin on their surface.	Streptavidin is immobilized on the test line to capture the biotin exposed on the GNPs. The availability of biotin on the GNP surface is dependent on the presence of the target analyte.	The novelty is related to the on-particle CHA enzyme-free signal amplification combined with the LFA platform	GNP modification takes a long time, and the method is not applicable in blood plasma	miR-21	0.89 pM	[58]
The integration of a tetrahedral probe and a catalytic hairpin assembly involves labeling DNA tetrahedrons with biotin to serve as capture probes on the test line. The catalytic hairpin assembly system consists of two hairpin sequences that form double-stranded products through an assembly reaction triggered by the target analyte.	The hybridized products, containing barcoded tetrahedra that utilize complementary sequences, bind to streptavidin-modified GNPs via biotin and are captured on the test line, where streptavidin is immobilized.	The novelty is related to the strategy for fabricating a strip-sensing interface based on the DNA tetrahedron structure for solving the restrictions for tethering nucleic acid probes with protein to avoid self-movement	Amplification step is required	miR-150-5p	58.90 fM	[59]

### 2.2. Improving Sensitivity of LFA Visual Detection by Enhancing the GNP Analytical Signal

The widespread use of GNPs as labels for LFA has pushed research to find innovative solutions to increase the sensitivity of the analysis through modifications of GNP-based tracers. In particular, AuNP-coated silica nanorods [66], horseradish peroxidase (HRP)-coated AuNPs [67], and carbon nanotubes [68] have been proposed as colored labels to enhance the sensitivity of these assays.

Platinum nanoparticles and their derivatives have been extensively applied in bioanalytical applications because they show good biological compatibility, high stability, and high catalytic activity [69,70]. Recently, some works reporting the use of gold–platinum nanoflowers (AuPt NFs) and AuPt nanocatalysts have been proposed for different biomedical analyses [71,72,73,74].

**Figure 3:**
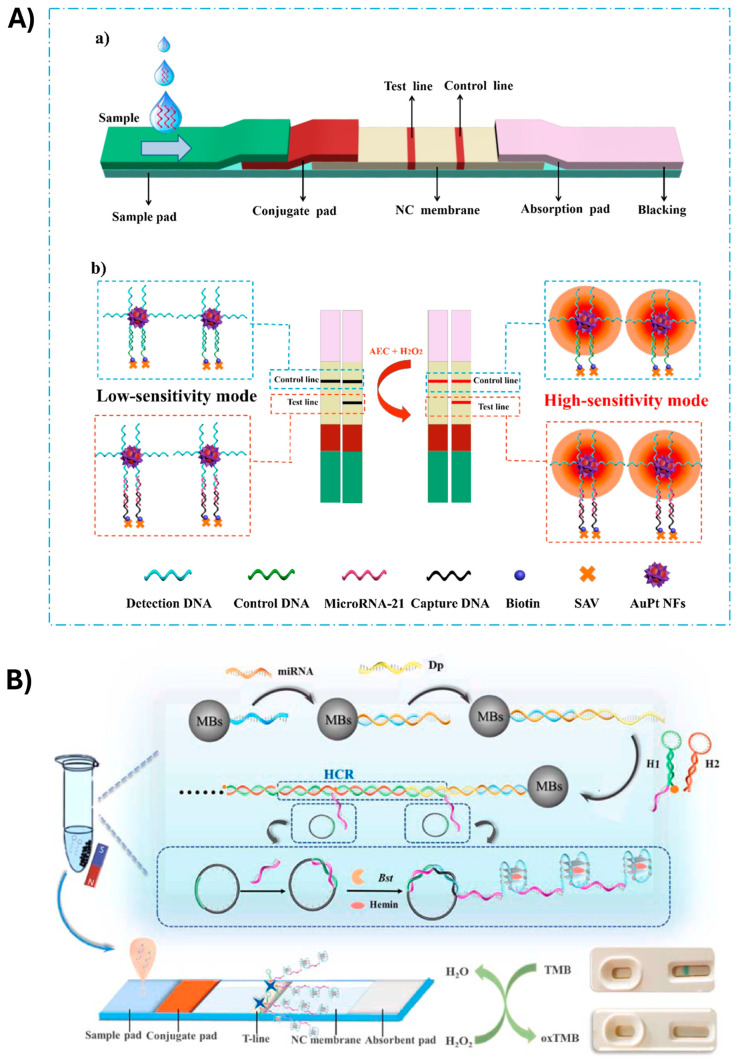
(**A**) Scheme of AuPt NF-based LFA: (**a**) the configuration of the assay; (**b**) enhancement of colorimetric signal through addition of the chromogenic substrate. Reprinted with permission from Ref. [75]. (**B**) Scheme of the reaction for the detection of miRNA based on HRCA reaction. Reprinted with permission from Ref. [85].

Zhang et al. [75] proposed the use of AuPt NFs for the sensitive detection of miR-21 (Figure 3A). AuPt NFs were obtained by loading Pt nanowires onto the outer layer of GNPs. A single-stranded DNA probe, complementary to the target miRNA-21, was conjugated with AuPt NFs to act as a detection probe. AuPt NFs were employed as a colored label for visual detection (through the formation of a black band when the target analyte was present) and as catalytic labels to allow quantitative analysis. The signal due to the catalytic activity of the label was triggered by the addition of a chromogenic substrate.

Also, Shi et al. [76] exploited a Pt-based label in order to enhance the sensitivity of the LFA. In particular, a platinum-coated gold nanoparticle (Au@PtNP) with catalase-like activity was proposed as a tracer to achieve a dual-signal readout. In this case, double detection was obtained visually through the formation of the colored band and via gas pressure biosensing. Indeed, Au@PtNP catalyzed the decomposition of H_2_O_2_, resulting in gas pressure changes measured by a digital handheld gas pressure meter.

As an alternative to Pt, silica has also been widely used to enhance GNP visual detection [66,77,78].

Takalkar et al. [37] exploited GNP-coated silica nanorods as a sensitive tracer for the detection of miR-215. A single-stranded DNA probe was labeled with gold nanoparticle–silica nanorod using a self-assembling procedure, and the formed DNA–gold nanoparticle–silica nanorod conjugate was used as a detection probe. The gold nanoparticle–silica nanorods, captured by sandwich-type hybridization reactions on the test line, produced the characteristic color bands, enabling the visual detection of miRNA.

Another approach was proposed by Wang et al. [79], who assembled gold nanoaggregates (AuNA) to amplify the signal for detecting TK1-miRNA. Four functional oligonucleotides with complementary sequences were assembled to form DNA–AuNA, which coupled more GNPs to improve sensitivity.

In conclusion, the extensive use of GNPs as labels in LFAs has driven significant advancements in improving the sensitivity of these techniques through innovative tracer modifications (Table 2). Strategies incorporating novel materials, such as platinum nanoparticles, silica, and hybrid nanocomposites, have shown great promise in enhancing detection capabilities. Approaches like AuPt NFs, Au@PtNPs, Au@Si nanocomposites, and DNA–gold nanoparticle assemblies have introduced dual readouts, catalytic amplification, and improved visual signals, contributing to more reliable and sensitive biomedical analyses.

### 2.3. Enzymatic Activity and G-Quadruplex Horseradish Peroxidase-Mimicking DNAzyme for Colorimetric Ultrasensitive Detection

The use of enzymes as optical labels for developing an ultrasensitive LFA technique has been extensively proposed for biomedical applications [80,81]. In this context, horseradish peroxidase (HRP) is one of the most commonly used enzymes as a label, thanks to its versatility, the ability to use different enzymatic substrates, and its ease of conjugation to biomolecules. Gao et al. [20] exploited HRP as a tracer for developing an LFA biosensor for detecting miR-224.

The detection DNA probe and HRP were immobilized on the GNP surface simultaneously. When miRNA-224 was present, the complex formed by the target analyte and detection probe was captured on the test line, where a sequence complementary to the target analyte was immobilized. By applying the 3,3,5,5-tetramethylbenzidine enzymatic substrate (TMB/H2O2 enzymatic substrate), blue products were obtained, enabling sensitive detection of the analyte of interest.

Recently, the use of a DNA structure able to mimic enzyme activity has been proposed, and several biosensing systems have been based on this approach [82,83,84].

In the field of LFA for miRNA detection, an example was reported by Li et al. [85]. The authors developed an LFA biosensor utilizing cascade nucleic acid amplification technology (HRCA) for the colorimetric detection of miR-31. The presence of miR-31 facilitated the formation of a sandwich structure on the surface of magnetic beads, which then triggered a cascade amplification reaction involving hybridization chain reaction (HCR) and rolling-circle amplification (RCA). This process generated G-quadruplex structures, which subsequently bound with hemin to form a hemin/G-quadruplex HRP-mimicking DNAzyme (H/G-HRP mimic enzyme). Captured on the test line (T-line), this enzyme catalyzed the oxidation of chromogenic substrates, producing a visible colorimetric signal on the strip (Figure 3B).

Another example of a colorimetric test based on enzymatic activity was reported by Wang et al. [86], who coupled the CRISPR/Cas13a system with MnO_2_ nanozymes for sensitive detection of miR-21. Upon binding of the target to crRNA, the cleavage activity of Cas13a was activated, resulting in the unlocking of the sequence and initiation of strand displacement, thereby enabling signal amplification to produce a new sequence, P1. When the reaction solution was applied to the LFA strip, P1 induced the capture of MnO_2_ nanosheets on the test line, which catalyzed the oxidation of the pre-immobilized colorless substrate TMB, thus enabling the generation of a blue-green product.

To summarize, the use of enzymes as optical labels has significantly advanced the development of ultrasensitive LFAs for biomedical applications (Table 2). Among these, HRP has emerged as a popular choice due to its versatility, broad substrate compatibility, and ease of conjugation with biomolecules. Innovative approaches, such as those by Gao et al. [20], demonstrate the potential of HRP-based tracers to enable sensitive and reliable detection of target analytes like miRNA. Additionally, enzyme-mimicking DNA structures, such as G-quadruplex-based HRP mimics, and hybrid systems incorporating nucleic acid amplification technologies (e.g., HRCA and HCR) have expanded the toolkit for enhancing colorimetric signal outputs. Emerging techniques like the integration of CRISPR/Cas13a systems with MnO_2_ nanozymes further highlight the potential for signal amplification and improved sensitivity. These advancements underline the transformative role of enzymatic and enzyme-mimicking systems in pushing the boundaries of LFA technology, making them indispensable tools in modern diagnostics.

**Table 2:** LFA-based biosensing systems for the quantification of miRNAs exploiting different strategies to enhance the performance of colorimetric detection.

Method Principle	Lateral Flow Assay	Biomarker	Lable	Key Strengths	Weaknesses and Areas Needing Improvement	LOD	Ref.
The detection utilized a sandwich approach, where the capture probe on the test line recognized the target analyte bound to a detection probe labeled with AuPt NFs.	A single-stranded DNA probe complementary to the target miRNA-21 was conjugated with AuPt NFs, serving as the detection probe. AuPt NFs functioned as both colored labels for visual detection, indicated by the formation of a black band when the target analyte was present, and as catalytic labels enabling quantitative analysis. The catalytic activity of the label was activated by adding a chromogenic substrate, generating the detectable signal.	miR-21	AuPt NFs	AuPt NFs were used both as colored and catalytic labels	The biosensor should be tested on real samples	0.3 pM	[75]
miRNAs served as the input to initiate the cyclic strand displacement reaction (SDR), producing a significant enrichment of Au@PtNPs. These nanoparticles enabled dual detection outputs. First, the grayish-brown color of Au@PtNPs provided a visual signal for miRNA detection via LFA (output 1). Second, the Au@PtNPs catalyzed the decomposition of H_2_O_2_, generating gas pressure measured using a digital handheld gas pressure meter (output 2).	During the SDR process, the target miRNA bound to biotin, and the detection probe conjugated with Au@PtNPs. As the complex flowed across the LFA membrane, it was captured on the test line by streptavidin.	miR-21	Au@PtNP	The novelty is related to the dual-mode logic gates based on gas pressure biosensing and LFA. Both color and gas pressure readouts were obtained	Amplification step is required	7.2 pM	[76]
The GNP-silica nanorod label was used to amplify the visual analytical signal.	A single-stranded DNA probe was labeled with gold nanoparticle–silica nanorods through a self-assembly process, and the resulting DNA–gold nanoparticle–silica nanorod conjugate served as the detection probe. The gold nanoparticle–silica nanorods were captured on the test line through sandwich-type hybridization reactions, generating characteristic color bands that enabled the visual detection of miRNA.	miR-215	GNP-silica nanorod	No complex sample treatment is needed	The method should be applied to detect miRNA in cell lysate and biological fluids	10 pM	[37]
Four functional oligonucleotides with complementary sequences were combined to form DNA-AuNA, which incorporated additional GNPs to enhance sensitivity.	In the sandwich approach, the detection probe linked to AuNA binds to the target analyte, and the resulting complex is captured by a complementary sequence immobilized on the test line.	TK1-miRNA	Gold nanoaggregate	Smartphone was used as detector	Low sensitivity of the visual assay	0.36 pM	[79]
HRP was used as a tracer and immobilized on the surface of GNPs, along with a detection DNA sequence complementary to the target analyte.	When miRNA-224 was present, the complex formed by the target analyte and detection probe was captured on the test line, where a complementary sequence to the target analyte was immobilized. Upon adding the 3,3,5,5-tetramethylbenzidine enzymatic substrate (TMB/H_2_O_2_), blue products were generated, enabling sensitive detection of the analyte of interest.	miR-224	HRP	Double detection	The biosensor could be applied for the detection of mismatched miRNA and multiplex miRNA in aqueous solutions and human fluids	7.5 pM	[20]
The combination of HCR and RCA was used to generate G-quadruplex structures, which could catalyze a colorimetric reaction in the presence of hemin.	When the target, miR-31, was present, it facilitated the formation of a sandwich structure on the surface of magnetic beads. This complex then triggered a cascade amplification reaction between hybridization chain reaction (HCR) and rolling-circle amplification (RCA), resulting in the formation of G-quadruplex structures. These structures, when combined with hemin, formed a hemin/G-quadruplex HRP-mimicking DNAzyme (H/G-HRP mimic enzyme), which was captured on the test line. The enzyme catalyzed the oxidation of chromogenic substrates, generating a colorimetric signal on the strip.	miR-31	G-quadruplex HRP-mimicking DNAzyme	High sensitivity due to the G-quadruplex structure approach for obtaining colorimetric detection	Amplification step is required	3.21 fM	[85]
The CRISPR/Cas13a system was integrated with MnO_2_ nanozyme in the design of an LFA biosensing strategy.	When the target bound to crRNA, the cleavage activity of Cas13a was activated, which unlocked the sequence and initiated strand displacement, leading to signal amplification and the production of a new sequence, P1. Upon applying the reaction solution to the LFA strip, P1 facilitated the capture of MnO_2_ nanosheets on the test line. These nanosheets then catalyzed the oxidation of the pre-immobilized colorless substrate 3,3′,5,5′-tetramethylbenzidine (TMB), resulting in the generation of a blue-green product.	miR-21	MnO_2_ nanosheets	The dual signal amplification mechanisms (CRISPR/Cas13a-mediated cleavage and the MnO_2_ NSs-catalyzed chromogenic reaction) increase sensitivity	Before the analysis with LFA, an incubation step at 37 °C is required	0.33 pM	[86]

## 3. Innovative Detection Approaches Combined with LFA Technology for miRNA Detection

Colorimetric detection based on the formation of colored bands on the membrane is the most widespread and simplest approach to be combined with LFA technology. However, research is moving in the direction of applying different detection strategies to increase the detectability of the analytes of interest, thus expanding the field of application of LFA systems. 

Starting with optical detection approaches, surface-enhanced Raman scattering (SERS) is one of the most widely employed analytical techniques [87,88,89] for the development of ultrasensitive portable biosensors. Indeed, narrow peaks, high selectivity, abundant fingerprint information, and water inactivity make SERS an optical technology that allows the detection of target analytes even down to the single-molecule level. Among the tracers that can be used for SERS-based detection, Au–Ag nanoshuttles (Au–AgNSs) have attracted attention due to their special gold–silver alloy structure, which provides improved chemical and physical stability, low synthesis costs, mild preparation conditions, and high SERS activity [90,91].

Cao et al. [38] proposed an SERS–LFA biosensor combined with CHA signal amplification for the quantification of miR-196a-5p and miR-31-5p. When the target miRNA was present, two hairpin DNAs self-assembled into double-stranded DNA, exposing biotin molecules on the surface of Au–AgNSs. SERS complexes were trapped on the T-lines, allowing SERS signals to be obtained.

The same approach was employed by Li et al. [92], who described an SERS–LFA biosensor based on the CHA amplification strategy for monitoring miR-106b and miR-196b. In this case, in the presence of the target, two hairpin DNAs self-assembled, forming double-stranded DNA and exposing the biotin molecules modified on the surface with a SERS tag that consisted of palladium (Pd)–gold (Au) core–shell nanorods (Pd–AuNRs). The biotin molecules were then captured by the streptavidin immobilized on the T-lines.

In this context, Dong et al. [93] reported the use of an Au@Si nanocomposite as a label for the dual detection (visual and based on the formation of an SERS signal) of miR-21. This label was obtained by coating GNPs on silica nanoparticles. The assay was based on the formation of a sandwich-like structure in which the label was bound to detection probes complementary to the target analyte, and this complex was then detected on the test line, where another complementary DNA sequence was immobilized.

As an alternative to Au-based conjugates, fluorescent labels, such as fluorescent microspheres and quantum dots, have attracted increasing interest in LFA techniques to obtain enhanced detection performance [94]. Since the advent of smartphones, these tools have been proposed as affordable equipment to integrate with portable biosensors, thanks to their high-quality cameras, microprocessors, and wireless communication capabilities [95,96], and this approach has also been exploited for fluorescent signal detection by implementing smartphones with external optical or electrical components to reduce complexity and enable miniaturization [97]. Applications were reported for nucleic acid amplification tests [98,99] and as detection strategies for LFA-based techniques [100,101,102]. In this context, He et al. [103] proposed the combination of rolling circle amplification with a fluorescent-LFA biosensing approach exploiting fluorescent microspheres (FMs) as tracers. The amplified products of the target miRNA and the FM probe conjugates formed complexes that ran along the LFA membrane. Then, the complexes specifically bound to the probes immobilized on the T-lines, and a positive signal (green fluorescence) could be obtained by the smartphone-based strip reader.

**Figure 4:**
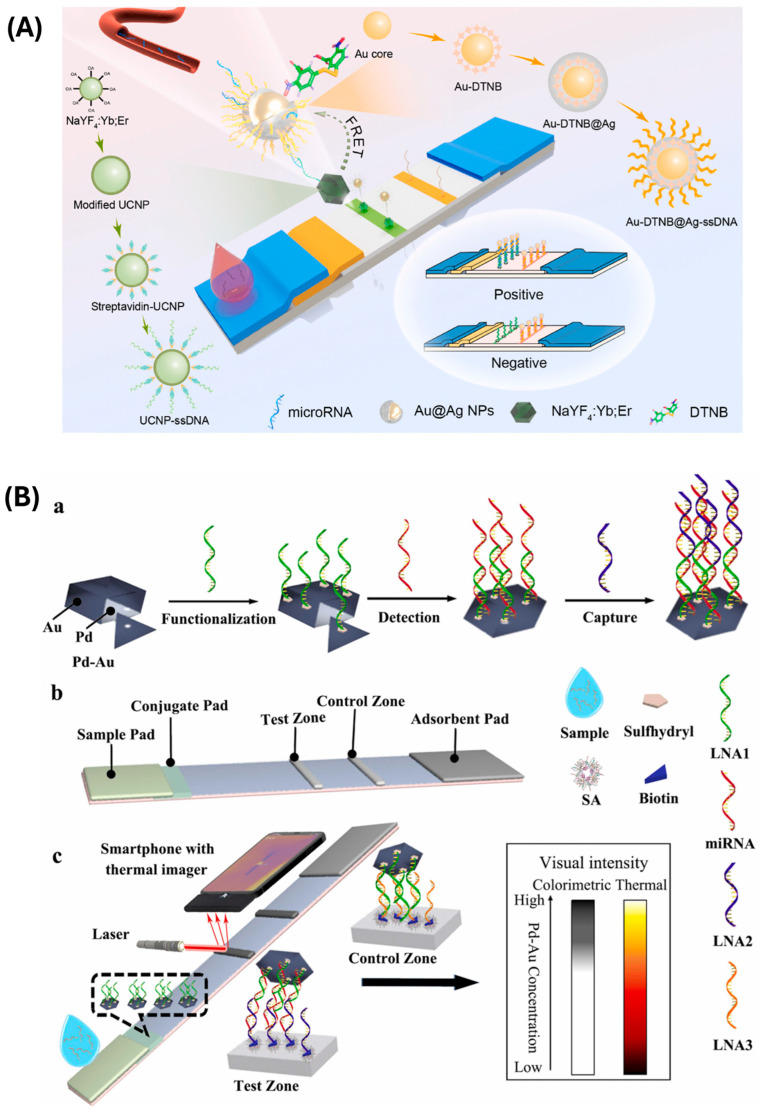
(**A**) The schematic diagram and synthetic route of the multimode lateral flow assay (LFA) biosensor for microRNA detection are shown. Upon adding the analyte to the strip, Au-DTNB@Ag NPs quench the fluorescence through the FRET process, leading to detectable changes in the fluorescence signal. Simultaneously, the colorimetric and SERS signals are enhanced as the Au-DTNB@Ag NPs accumulate on the T-line. The flowcharts on both sides detail the synthetic pathways of the nanoparticles. Reprinted with permission from Ref. [104]. (**B**) Schematic representation of (**a**) the preparation and functionalization of Pd-Au nanoplates and chain hybridization for miRNA detection; (**b**) the components; and (**c**) the construction of a P-LFLNAB, illustrating the relationship between colorimetric and thermal signals and the concentration of Pd-Au nanoplates. Reprinted with permission from Ref. [108].

Chen et al. [104] exploited the phenomenon of fluorescence quenching to develop an LFA-based system for the detection of miR-21 (Figure 4A). In particular, they proposed the concurrent surface enhancement of Raman scattering and fluorescence signal generation. Au-DTNB@Ag NPs acted as both Raman reporters and acceptors, enabling fluorescence quenching through the FRET mechanism when in proximity to metal particles. The drying process caused a narrowing of the gap between the donor (UCNPs) and the acceptor (DTNB@Ag NPs), making the signal detectable by the fluorescent detector. The use of these gold-based NPs, in addition to providing fluorescence quenching and acting as Raman reporters, also enabled the detection of the SERS signal. Indeed, thanks to the traditional visible color changes in the test and control zones, it was also possible to obtain a visual detection for a qualitative interpretation, even by non-experts.

As an alternative to fluorescence, the NIR field has also been used, as the required external power source offers the advantages of excellent biocompatibility and remote manipulation [105,106]. Chen et al. [107], to overcome the problem of inefficient capture of miRNAs from complex biological samples, proposed the use of Janus nanomotors powered by NIR irradiation. These nanomotors were composed of Au nanorods and periodic mesoporous organosilica microspheres (AuNR/PMO JNMs) exploited as “swimming probes” to perform LFA for amplification-free quantification of miRNA-21 in serum and cell medium.

The AuNR/PMO JNMs were conjugated with an ad hoc-designed DNA recognition probe. Under NIR irradiation, the AuNRs generated asymmetric thermal gradients around the JNMs to obtain thermophoretic motion. The active movement accelerated the recognition of miRNA-21 targets, improving capture efficiency. The detection was performed with both visual and thermal signals.

Also, Huang et al. [108] proposed the use of the photothermal approach to develop an LFA-based biosensor for the detection of miR-21 (Figure 4B). The detection probes were based on the use of Pd–Au bimetallic nanoplates, which show photothermal properties conjugated to the specific sequence for binding the target analyte and preloaded on the conjugate pad. The capture probe for the recognition of miR-21 exploited the biotin–streptavidin interaction for immobilization on the T-line. Through the formation of a sandwich structure in the presence of the target analyte, it was possible to achieve both visual and thermal detection by employing a smartphone equipped with an external thermal imager and a portable laser for the irradiation of the conjugate probes.

To conclude, colorimetric detection, relying on the formation of colored bands on the membrane, remains the most widely used and simplest approach integrating LFA technology. However, research is advancing toward exploring diverse detection strategies to enhance sensitivity and expand the application range of LFA systems (Table 3). Among the emerging techniques, SERS is gaining prominence due to its ability to detect target analytes even at the single-molecule level. SERS-based LFA biosensors, such as those using Au–Ag nanoshuttles, offer improved stability, high activity, and cost-effectiveness, enabling highly sensitive analysis. Additionally, the integration of fluorescent labels, such as fluorescent microspheres and quantum dots, with smartphone-based systems presents an innovative approach to further enhance detection capabilities. Furthermore, alternative technologies, such as NIR and photothermal-based methods, have shown potential in providing efficient and highly accurate detection with minimal sample preparation.

**Table 3:** LFA-based biosensing systems for the quantification of miRNAs exploiting innovative detection approaches.

Method Principle	Lateral Flow Assay	Biomarker	Label	Key Strengths	Weaknesses and Areas Needing Improvement	LOD	Ref.
The SERS–LFA biosensor is combined with CHA signal amplification.	The detection of the target miRNA was performed by allowing two hairpin DNAs to self-assemble into double-stranded DNA, exposing biotin molecules on the surface of Au–AgNSs. The SERS complexes were then captured on the T-line, enabling the generation of SERS signals.	miR-196a-5p and miR-31-5p	Au–Ag nanoshuttles	Multiplex approach tested in human serum	The detection step required the acquisition of spectra, and the detector should be equipped with an excitation source	1.171 nM (miR-196a-5p) 2.251 nM (miR-31-5p)	[38]
SERS–LFA biosensor combined with CHA signal amplification.	In the presence of the target, two hairpin DNAs self-assemble into double-stranded DNA, exposing the biotin molecules modified with an SERS tag on the surface.	miR-106b and miR-196b	palladium (Pd)–gold (Au) core–shell nanorods	Pd–AuNRs were easily synthesized on a large scale, and this label allowed very strong SERS signals	The detection step required the acquisition of spectra, and the detector should be equipped with an excitation source	23.17 aM (miR-106b) 46.94 aM (miR-196b)	[92]
The Au@Si nanocomposite label was used to amplify the visual detectability and to obtain the formation of SERS signals.	The assay relied on a sandwich-like structure, where the label bound to detection probes complementary to the target analyte. This complex was then captured on the test line, where a complementary DNA sequence was immobilized.	miR-21	Au@Si	No complex sample treatment is needed	The method could be applied for multiplex assay, and it should be tested in serum samples from patients and healthy controls	1 pM	[93]
Combination of RCA and FL-LFA.	The amplified products of the target miRNA and the FM–probe conjugates formed complexes that traveled along the LFA membrane. These complexes were then specifically captured by the probes immobilized on the T-line, and a positive green fluorescence signal was obtained using the smartphone-based strip reader.	miRNA 21 and miRNA let-7a as low as	Fluorescent microspheres	Smartphone-based strip reader was successfully designed and fabricated for measuring the fluorescence signals	Amplification step is required	230.60 pM (miRNA 21) 27.89 pM (miRNA let-7a)	[103]
Amplification-free LFA based on Janus nanomotors powered by NIR irradiation.	Au nanorods and periodic mesoporous organosilica microspheres (AuNR/PMO JNMs) were utilized to develop nanomotors functioning as “swimming probes.” These AuNR/PMO JNMs were conjugated with custom-designed DNA recognition probes. Under NIR irradiation, the AuNRs generate asymmetric thermal gradients around the JNMs, inducing thermophoretic motion. This active movement enhances the recognition of miRNA-21 targets, thereby improving capture efficiency.	miR21	AuNR/PMO JNMs	The active movement accelerated the recognition of the target	The processes were divided into the hybridization step (which required irradiation by NIR light for 10 min) and strip detection procedure (which required recording the thermal signals at the T-line under excitation of NIR light for 1 min by an electronic thermometer)	18 fmol/L	[107]
Photothermal approach based on the use of bimetallic particles as tracers for simultaneously obtaining visual and quantitative information using a smartphone as a detector.	The detection probes were based on Pd–Au bimetallic nanoplates, which exhibit photothermal properties and are conjugated to a specific sequence for binding the target analyte. These probes were pre-loaded onto the conjugate pad. The capture probe, designed to recognize miR-21, utilized the biotin–streptavidin interaction for immobilization on the T-line. Upon formation of the sandwich structure in the presence of the target analyte, both visual and thermal detection were achieved using a smartphone equipped with an external thermal imager and a portable laser for irradiating the conjugate probes.	miR-21	Pd-Au nanoplates	The biosensor achieved single-base mismatch discrimination and could quantify signals in various cell lysates	The equipment is quite complex because a laser is needed to irradiate the test line, and the thermal image is captured by the smartphone with an external thermal imager	0.094 pM	[108]

## 4. Discussion

Early non-invasive point-of-care detection, diagnosis, and prognosis of cancer are critical for reducing mortality, morbidity, and treatment costs. Consequently, there is an urgent need to identify novel biomarkers and develop rapid, low-cost diagnostic tools. In this context, the potential of miRNAs as biomarkers for cancer diagnosis and disease monitoring opens new avenues in clinical analytical chemistry. Their stability in biological fluids, differential expression patterns in various cancers, and ability to reflect disease states make them highly valuable for early cancer detection.

miRNAs play a significant role in various cellular processes, such as proliferation, transcription, differentiation, and apoptosis. These biomolecules can provide valuable insights into the initiation and progression of diseases, particularly cancer. Aberrant expression of specific miRNAs has been linked to tumorigenesis, metastasis, and drug resistance, underscoring their clinical importance. Moreover, the presence of miRNAs in biological fluids such as blood, saliva, and urine makes them excellent biomarkers for detecting cancer at very early stages, often before clinical symptoms appear. The non-invasive nature of miRNA detection presents a significant advantage over traditional biopsy-based diagnostic methods.

Given the clinical importance of miRNAs and the limitations of existing analytical techniques for their quantification, the development of an effective LFA-based system offers a promising solution. LFA is a widely used POC diagnostic method due to its simplicity, affordability, rapid turnaround time, and user-friendly nature. These systems enable easy, sensitive, and rapid detection of disease-specific miRNAs, facilitating early diagnosis and timely treatment decisions. As cancer treatments are more effective when administered in the early stages of the disease, an LFA-based detection system for miRNAs could significantly improve patient outcomes by enabling timely intervention.

The adoption of LFA-based POC devices for miRNA detection is expected to grow significantly in the coming years. LFA is a cost-effective method that has already demonstrated versatility, particularly in healthcare applications, including infectious disease diagnostics and pregnancy tests. Recent innovations in the LFA platform, such as the incorporation of nanomaterials and ultrasensitive tracers, have further enhanced its ability to provide rapid and specific disease diagnoses. These devices do not require specialized laboratory equipment, making them accessible even in remote or underdeveloped areas. This accessibility is crucial for improving global healthcare equity, particularly in low-resource settings where advanced diagnostic technologies are often unavailable.

A review of recent literature on analytical devices using the LFA method for miRNA detection reveals trends similar to those seen in immunoassay-based LFAs for clinical applications. For example, GNPs are commonly used as tracers for semi-quantitative analyte detection due to their strong optical properties, which enable easy visualization [40,45,46,47,48,49,51,52,53,54,55,56,57,58,59]. However, an increasing number of studies propose alternative tracers, such as quantum dots [103] and enzyme-based [20,85] amplification strategies, which enable signal amplification and ultra-sensitive quantification of analytes. Sensitivity is a critical feature, as these methods aim to diagnose diseases at their earliest stages. As summarized in the tables, the developed methods achieve high sensitivities, generally in the picomolar (pM) range, with some reaching femtomolar (fM) levels, highlighting the significant advancements in LFA technology.

In some cases, amplification steps such as polymerase chain reaction (PCR) or rolling circle amplification (RCA) are required before using the LFA method [45,46,49,51,52,55,56,57,58], which can complicate the procedure or limit its large-scale application outside of centralized laboratories. These amplification steps, while beneficial for enhancing sensitivity, introduce additional costs and technical requirements that may not be feasible in POC settings. Biosensors that eliminate these additional steps have shown promising results, combining high sensitivity with ease of use. However, integrating LFA systems with cost-effective and user-friendly detectors for quantitative analysis remains a challenge. With the rapid advancement of compact, high-performance devices, such as smartphone-based readers and miniaturized fluorescence detectors, these hurdles are expected to be resolved in the near future, enabling seamless integration of LFA technology into routine clinical practice.

Innovative tracers and biospecific recognition methods are also expected to yield increasingly sensitive and efficient systems. Aptamers, molecular beacons [53,54,55], and CRISPR-based detection strategies [65,86] are being explored to further improve the specificity and efficiency of miRNA detection. Validation of miRNA biomarkers remains a critical challenge, as their expression levels vary depending on the type and stage of the disease. Moreover, external factors such as sample collection, storage, and processing can influence miRNA stability, necessitating standardized protocols for accurate and reproducible results.

Simultaneous analysis of multiple miRNAs could significantly improve early disease diagnosis, as single miRNA data are often insufficient to capture the complexity of cancer progression. Multiplexed LFA systems that allow parallel detection of multiple miRNAs in a single assay are being developed to address this limitation [38,40,46,48,49,51,52,54,92,103]. Additionally, big data analysis techniques, including machine learning and artificial intelligence, could play a pivotal role in correlating multiple parameters, further enhancing diagnostic accuracy. By integrating large-scale datasets from diverse patient populations, AI-driven diagnostic models can improve the sensitivity and specificity of miRNA-based assays, ultimately leading to more personalized and precise cancer diagnostics.

To summarize, LFA-based miRNA detection represents a promising approach for the early diagnosis and monitoring of cancer. Advances in nanotechnology, biosensor development, and data analytics are driving the evolution of these diagnostic tools, making them more sensitive, specific, and accessible. While challenges such as biomarker validation, assay reproducibility, and integration with quantitative detection systems remain, ongoing research and technological innovations are expected to address these issues. The continued development of LFA-based miRNA detection systems has the potential to revolutionize cancer diagnostics, offering a rapid, cost-effective, and non-invasive solution that could significantly improve patient outcomes worldwide. 

## 5. Conclusions

MicroRNAs represent a promising class of biomarkers for early cancer diagnosis due to their stability, specificity, and association with multiple types of tumors. The ability to detect changes in miRNA levels even during the early stages of the disease makes them a critical diagnostic tool for improving prognosis and the effectiveness of cancer therapies.

In this context, lateral flow assay (LFA)-based methods emerge as ideal solutions for the rapid quantification of miRNAs, offering significant advantages over conventional techniques. LFAs combine simplicity, sensitivity, and portability, enabling low-cost analyses that do not require centralized laboratories or highly specialized personnel. These features make them particularly suitable for frequent and large-scale screening applications, fostering more inclusive and widespread access to oncological diagnostics.

The future development of LFA-based devices for miRNA quantification lies in the continuous optimization of materials and detection strategies to further enhance their sensitivity, specificity, and multiplexing capabilities. Integrating innovative technologies, such as advanced nanomaterials and signal amplification systems, could further boost the performance of these tools, solidifying their role in personalized medicine and cancer prevention.

In conclusion, LFA methods for miRNA detection represent a crucial step toward the development of accessible, non-invasive, and reliable diagnostic systems, significantly contributing to advancements in early cancer diagnosis and management.

## Data Availability

Not applicable.
